# *Haemophilus influenzae* Type a Meningitis in Immunocompetent Child, Oman, 2015

**DOI:** 10.3201/eid2307.170311

**Published:** 2017-07

**Authors:** Kiran P. Sawardekar

**Affiliations:** Nizwa Hospital, Nizwa, Sultanate of Oman

**Keywords:** Haemophilus influenzae, Haemophilus influenzae serotype a, non-b Haemophilus influenzae, meningitis/encephalitis, invasive non-b Haemophilus influenzae disease, Oman, bacteria

## Abstract

Meningitis caused by *Haemophilus influenzae* type b (Hib) was eliminated in Oman after the introduction of Hib vaccine in 2001. However, a case of *H. influenzae* type a meningitis was diagnosed in a child from Oman in 2015, which highlights the need to monitor the incidence of invasive non-Hib *H. influenza*e disease.

*Haemophilus influenzae* can be encapsulated (serotypes a–f) or unencapsulated, nontypeable (NTHi) (*1*). By the end of 2014, all countries in the Eastern Mediterranean Region had introduced *H. influenzae* type b (Hib) vaccine into their immunization programs; in Oman, where it was introduced in 2001, it led to an elimination of Hib meningitis (*2**,**3*). However, Hib vaccine does not cross-protect against other serotypes.

A previously healthy 17-month-old girl with G6PD deficiency was admitted to Nizwa Hospital, Nizwa, Oman, in August 2015 with a 1-day history of fever and lethargy and frequent vomiting and refusal of food for 6–8 hours before admission. She had no history of rash, head trauma, drug ingestion, travel abroad, or contact with animals. Her vaccination record was up to date. Her 3 older siblings were healthy. On examination, she was irritable and febrile (temperature 39°C), with tachypnea, tachycardia, and photophobia. On lung auscultation, a few crackles were heard on the right side. The rest of her physical examination, including a bedside undilated fundoscopic examination, was unremarkable. Blood tests, cerebrospinal fluid examination, and neuroimaging studies were conducted (Table). Results of renal and liver function and metabolic screening tests and serum calcium, troponin T, immunoglobulins, and total complement levels were within reference limits. Diagnostic test results were negative for respiratory viruses including influenza A(H1N1) and Middle East respiratory syndrome coronavirus and for herpes simplex virus types 1 and 2. A chest radiograph showed right middle lobe haziness suggestive of pneumonitis.

**Table:** Results of sequential laboratory tests and CT scans of the head during the clinical course of Hia meningitis in a child admitted to Nizwa Hospital, Oman, August 2015*

Test type	Hospitalization day								
	1		2	8	10	14	18	26	27
	Adm	Adm + 12							
Blood									
Leukocytes, × 10^3^ cells/μL†	2.24	8.71	10.52	13.08		12.32	8.48	4.56	
Neutrophils, × 10^3^ cells/μL‡	0.78	6.04	7.66	4.92		5.51	2.50	0.82	
Lymphocytes, × 10^3^ cells/μL§	1.22	2.02	2.38	5.98		5.21	5.12	2.89	
Platelets, × 10^3^/μL¶	158.30	79.12	41.13	419.90		644.70	525.80	279.20	
Hemoglobin, g/dL#	10.18	9.30	9.13	9.06		8.47	9.22	10.38	
CRP, mg/L**	79.30		483.40	248.50			33.70	35.30	
Blood culture	Hia			NG				NG	
CSF									
Leukocytes, cells/mm^3^	2,970				390			65	
Neutrophils, %	91				10			12	
Lymphocytes, %	9				90			88	
Protein, mg/dL††	190.79				100.34			27.11	
Glucose, mmol/L‡‡	1.13				2.24			2.83	
Glucose CSF:blood	0.26				0.56			0.70	
Gram stain	NM				NM			NM	
Culture	NG				NG			NG	
CT scan of the head									
	Unremarkable			Mild ventricular dilation with bilateral subdural effusion					Complete disappearance of subdural effusion; persistence of mild ventricular dilation

*Adm, at admission; Adm + 12, 12 h after admission; CRP, C-reactive protein; CSF, cerebrospinal fluid; CT, computed tomography; Hia, *Haemophilus Influenzae* type a; NA, not applicable; NG, no growth; NM, no microorganisms. †Reference range 4.5–14.5 × 10^3^ cells/μL. ‡Reference range 1.4–9.0 × 10^3^ cells/μL. §Reference range 1.9–9.8 × 10^3^ cells/μL. ¶Reference range 150–450 × 10^3^/μL. #Reference range 11.5–15.5 g/dL. **Reference range 0–5 mg/L. ††Reference range 15–45 mg/dL. ‡‡Reference range 2.2–3.9 mmol/L.

The patient was treated with intravenous ceftriaxone. Blood culture revealed *H. influenzae* type a (Hia), which was serotyped by slide agglutination and determined to be β-lactamase negative, with susceptibility to all tested antimicrobial drugs. The patient had a protracted clinical course, characterized by continued photophobia, intermittent fever (38–39°C), and subdural effusion. After 10 days of ceftriaxone treatment, her drug therapy was changed to intravenous ampicillin, administered for 2 weeks. Her condition gradually improved; she became afebrile by day 21 after admission and was well at discharge on day 27. Results of vision and hearing screening tests 1 month after discharge and 1 year later were unremarkable.

Several case studies have documented prolonged clinical courses of Hia meningitis, with sequelae reported in some children (*4**,**5*; Technical Appendix Table). Hia meningitis is strikingly reminiscent of Hib meningitis, manifesting as a serious illness mostly in otherwise healthy children 6–24 months of age (*1*,*4**,**5*). Hia has been reported to be the most virulent among encapsulated *H. influenzae* after Hib; the genetic structure of virulent Hia strains closely resembles that of virulent Hib strains with respect to the duplicated arrangement of the capsule locus and, in some cases, partial deletion of the IS*1016-bexA* gene locus (*5**–**7*; Technical Appendix Table). An active hospital-based surveillance study for meningitis during 1996–2007 in Salvador, Brazil, reported that Hia and Hib meningitis occurred mainly among children <5 years of age; case-fatality rates were higher than those for meningitis caused by types e and f and NTHi strains, which occurred in older age groups and tended to have a better prognosis (*6*). The study observed an association between IS*1016-bexA* deletion and poor clinical outcome of Hia meningitis.

Since Hib vaccine implementation, concerns have arisen about serotype replacement and emergence of virulent non-b *H. influenzae* (*5**,**6*; Technical Appendix Table). With documentation of 3 cases (including the case reported here) of Hia meningitis in the Eastern Mediterranean Region within <2 years (*8*), more than a decade after Hib vaccine implementation, it is crucial to monitor meningitis in children within the region, complemented by laboratory characterization of incoming specimens by molecular methods for rapid, accurate information on all *H. influenzae* serotypes and NTHi (*1*; https://www.cdc.gov/meningitis/lab-manual/full-manual.pdf; Technical Appendix Table). In Oman, it is mandatory to report cases of Hib meningitis within 24 hours of laboratory diagnosis, and those caused by other serotypes and NTHi within 1 week, to the Department of Communicable Disease Surveillance and Control, Ministry of Health. Evidence of capsule-deficient variants of Hia that cannot be differentiated from NTHi by conventional methods (*7*) and recurrent invasive diseases (*9**,**10*) and outbreaks caused by Hia (*9*; Technical Appendix Table) emphasize the necessity for continued surveillance, strong laboratory support, and local epidemiologic studies on non-b *H. influenzae* disease.

Hia meningitis has been reported mainly in the indigenous peoples of Canada, Alaska (USA), and Australia; in the Navajo and White Mountain Apache tribes in the southwestern United States; and in Utah (USA), Brazil, the Gambia, East Africa, and Papua New Guinea. Sporadic cases have been reported in the rest of the world (*1**,**10*; Technical Appendix Table). The reasons behind the high rates of invasive Hia disease among indigenous children remain unclear (*1*). In Canada, where invasive non-b *H. influenzae* disease has been included in the list of nationally reportable diseases (http://diseases.canada.ca/notifiable/diseases-list) since 2007, a public health–driven initiative has been established to provide a better characterization of the epidemiology of invasive Hia disease and develop a candidate vaccine against Hia (Technical Appendix Table).

Technical Appendix Additional research on non–serotype b *Haemophilus influenzae*.
