# Supplementary material for: Haemophilus influenzae Type a Meningitis in Immunocompetent Child, Oman, 2015
Source: Emerg Infect Dis. 2017 Jul;23(7):1221–3. doi: 10.3201/eid2307.170311 (PMC5512487; doi:10.3201/eid2307.170311)
Supplement: Technical Appendix — Additional research on non–serotype b Haemophilus influenzae. [file 17-0311-Techapp-s1.pdf]

Article DOI: http://dx.doi.org/10.3201/eid2307.170311

# *Haemophilus influenzae* Type a Meningitis in Immunocompetent Child, Oman, 2015

## Technical Appendix

**Technical Appendix Table.** Additional research on non–serotype b *Haemophilus influenzae* (Hi)

| Authors | Year published | Location of study | Subject | Reference |
|---|---|---|---|---|
| Sadeghi-Aval P, et al. | 2013 | Northwestern Ontario, Canada | Non-Hib pediatric meningitis | *1* |
| de Pádua RA, et al. | 2009 | Paraná, Brazil | Hia meningitis | *2* |
| Kroll JS, et al. | 1994 | The Gambia | Virulence-enhancing mutation in Hia | *3* |
| Kapogiannis BG, et al. | 2005 | United States of America | IS1016-bexA partial deletion in Hia | *4* |
| Mulder DC, et al. | 2002 | The Netherlands | Hia meningitis in infant | *5* |
| Wang X, et al. | 2011 | Mongolia | A new real-time PCR to detect Hi | *6* |
| WHO, CDC | 2011 | | Laboratory methods | *7* |
| Bruce MG, et al. | 2013 | Alaska, USA | Invasive Hia disease | *8* |
| Boisvert AA, et al. | 2015 | North Canada | Invasive Hia disease in children | *9* |
| Greenhill AR, et al. | 2015 | Papua New Guinea | Pre-vaccine serotype distribution | *10* |
| Gounder PP, et al. | 2015 | North American Arctic | Bacterial meningitis | *11* |
| Desai S, et al. | 2015 | Ontario, Canada | Invasive non-Hib disease | *12* |
| Wan Sai Cheong J, et al. | 2015 | Queensland, Australia | Invasive *H. influenzae* disease | *13* |
| Tsang RS, et al. | 2016 | Nunavut, Canada | Invasive *H. influenzae* disease | *14* |
| Tuyama M, et al. | 2017 | Rio de Janeiro, Brazil | Invasive *H. influenzae* disease | *15* |
| Tsang RS, et al. | 2017 | Nunavik, Canada | Invasive Hia disease | *16* |
| Whittaker R, et al. | 2017 | Europe | Invasive *H. influenzae* disease | *17* |
| Efron AM, et al. | 2013 | Argentina | Post-vaccine serotype distribution | *18* |
| Desai S, et al. | 2014 | Canada | Vaccine development initiative | *19* |

### References

1. Sadeghi-Aval P, Tsang RS, Jamieson FB, Ulanova M. Emergence of non–serotype b encapsulated *Haemophilus influenzae* as a cause of pediatric meningitis in northwestern Ontario. Can J Infect Dis Med Microbiol. 2013;24:13–6. http://dx.doi.org/10.1155/2013/828730

2. de Pádua RA, de Lima Scodro RB, Ghiraldi LD, Siqueira VL, Yamashita YK, Helbel C, et al. *Haemophilus influenzae* serotype a meningitis. Ann Clin Lab Sci. 2009;39:405–8.

3. Kroll JS, Moxon ER, Loynds BM. Natural genetic transfer of a putative virulence-enhancing mutation to *Haemophilus influenzae* type a. J Infect Dis. 1994;169:676–9. http://dx.doi.org/10.1093/infdis/169.3.676

4. Kapogiannis BG, Satola S, Keyserling HL, Farley MM. Invasive infections with *Haemophilus influenzae* serotype a containing an IS*1016-bexA* partial deletion: possible association with virulence. Clin Infect Dis. 2005;41:e97–103. http://dx.doi.org/10.1086/498028

5. Mulder DC, Padberg RD, Westra M, Fijen CA. *Haemophilus influenzae* type a as the causative agent of meningitis in an infant [in Dutch]. Ned Tijdschr Geneeskd. 2002;146:1651–3.

6. Wang X, Mair R, Hatcher C, Theodore MJ, Edmond K, Wu HM, et al. Detection of bacterial pathogens in Mongolia meningitis surveillance with a new real-time PCR assay to detect *Haemophilus influenzae*. Int J Med Microbiol. 2011;301:303–9. http://dx.doi.org/10.1016/j.ijmm.2010.11.004

7. World Health Organization, Centers for Disease Control and Prevention. Laboratory methods for the diagnosis of meningitis caused by *Neisseria meningitidis*, *Streptococcus pneumoniae* and *Haemophilus influenzae*. WHO Manual. 2nd ed. 2011 [cited 2017 March 5]. https://www.cdc.gov/meningitis/lab-manual/full-manual.pdf

8. Bruce MG, Zulz T, DeByle C, Singleton R, Hurlburt D, Bruden D, et al. *Haemophilus influenzae* serotype a invasive disease, Alaska, USA, 1983–2011. Emerg Infect Dis. 2013;19:932–7. http://dx.doi.org/10.3201/eid1906.121805

9. Boisvert AA, Moore D. Invasive disease due to *Haemophilus influenzae* type a in children in Canada's north: a priority for prevention. Can J Infect Dis Med Microbiol. 2015;26:291–2. http://dx.doi.org/10.1155/2015/613820

10. Greenhill AR, Phuanukoonnon S, Michael A, Yoannes M, Orami T, Smith H, et al. *Streptococcus pneumoniae* and *Haemophilus influenzae* in paediatric meningitis patients at Goroka General Hospital, Papua New Guinea: serotype distribution and antimicrobial susceptibility in the pre-vaccine era. BMC Infect Dis. 2015;15:485. http://dx.doi.org/10.1186/s12879-015-1197-0

11. Gounder PP, Zulz T, Desai S, Stenz F, Rudolph K, Tsang R, et al. Epidemiology of bacterial meningitis in the North American Arctic, 2000–2010. J Infect. 2015;71:179–87. http://dx.doi.org/10.1016/j.jinf.2015.04.001

12. Desai S, Jamieson FB, Patel SN, Seo CY, Dang V, Fediurek J, et al. The epidemiology of invasive *Haemophilus influenzae* non–serotype b disease in Ontario, Canada from 2004 to 2013. PLoS One. 2015;10:e0142179. http://dx.doi.org/10.1371/journal.pone.0142179

13. Wan Sai Cheong J, Smith H, Heney C, Robson J, Schlebusch S, Fu J, et al. Trends in the epidemiology of invasive *Haemophilus influenzae* disease in Queensland, Australia from 2000 to 2013: what is the impact of an increase in invasive non-typable *H. influenzae* (NTHi)? Epidemiol Infect. 2015;143:2993–3000. http://dx.doi.org/10.1017/S0950268815000345

14. Tsang RS, Li YA, Mullen A, Baikie M, Whyte K, Shuel M, et al. Laboratory characterization of invasive *Haemophilus influenzae* isolates from Nunavut, Canada, 2000–2012. Int J Circumpolar Health. 2016;75:29798. http://dx.doi.org/10.3402/ijch.v75.29798

15. Tuyama M, Corrêa-Antônio J, Schlackman J, Marsh JW, Rebelo MC, Cerqueira EO, et al. Invasive *Haemophilus influenzae* disease in the vaccine era in Rio de Janeiro, Brazil. Mem Inst Oswaldo Cruz. 2017;112:196–202. http://dx.doi.org/10.1590/0074-02760160391

16. Tsang RS, Proulx JF, Hayden K, Shuel M, Lefebvre B, Boisvert AA, et al. Characteristics of invasive *Haemophilus influenzae* serotype a (Hia) from Nunavik, Canada and comparison with Hia strains in other North American Arctic regions. Int J Infect Dis. 2017;57:104–7. http://dx.doi.org/10.1016/j.ijid.2017.02.003

17. Whittaker R, Economopoulou A, Dias JG, Bancroft E, Ramliden M, Celentano LP; European Centre for Disease Prevention and Control Country Experts for Invasive Haemophilus influenzae Disease. Epidemiology of invasive *Haemophilus influenzae* disease, Europe, 2007–2014. Emerg Infect Dis. 2017;23:396–404. http://dx.doi.org/10.3201/eid2303.161552

18. Efron AM, Moscoloni MA, Reijtman VR, Regueira M. Surveillance of *Haemophilus influenzae* serotypes in Argentina from 2005 to 2010 during the *Haemophilus influenzae* type b conjugate vaccine era [in Spanish]. Rev Argent Microbiol. 2013;45:240–7. http://dx.doi.org/10.1016/S0325-7541(13)70030-0

19. Desai S, Tsang R, St. Laurent M, Cox A. Collaboration on a public health–driven vaccine initiative. Can Commun Dis Rep. 2014;40:365–8.
